# Supplementary material for: Detailed molecular and epigenetic characterization of the pig IPEC-J2 and chicken SL-29 cell lines
Source: iScience. 2023 Feb 20;26(3):106252. doi: 10.1016/j.isci.2023.106252 (PMC10018572; doi:10.1016/j.isci.2023.106252)
Supplement: Data S2. Complete homer output for identified motifs in Chicken SL-29, related to Tables 5 and 6 — Homer motif analysis results for histone modifications H3K4me1, H3K4me3, H3K27ac, enhancers, and ATAC-seq of chicken SL-29 cell line. Parameters for possible false positives is as mentioned earlier for S5. [file mmc3.zip › Data_S2/S6/Chicken_SL_29/peak_files_H3K4me1/homerResults/motif5.similar.html]

motif5

## Information for motif5

G
A
T
C
G
A
C
T
A
G
T
C
C
G
T
A
A
C
T
G
A
G
T
C
G
A
T
C
G
A
T
C
G
T
A
C
A
C
G
T
A
T
G
C
C
T
A
G
A
G
C
T
  
Reverse Opposite:  

C
T
G
A
A
G
T
C
T
A
C
G
C
G
T
A
A
C
T
G
C
A
T
G
C
T
A
G
A
C
T
G
A
G
T
C
C
G
A
T
T
A
C
G
C
G
T
A
C
A
T
G
  

|  |  |
| --- | --- |
| p-value: | 1e-35 |
| log p-value: | -8.254e+01 |
| Information Content per bp: | 1.853 |
| Number of Target Sequences with motif | 32.0 |
| Percentage of Target Sequences with motif | 0.06% |
| Number of Background Sequences with motif | 0.0 |
| Percentage of Background Sequences with motif | 0.00% |
| Average Position of motif in Targets | 144.8 +/- 88.9bp |
| Average Position of motif in Background | 0.0 +/- 0.0bp |
| Strand Bias (log2 ratio + to - strand density) | 0.2 |
| Multiplicity (# of sites on avg that occur together) | 1.00 |
| Motif File: | file (matrix) reverse opposite |

### Similar de novo motifs found

|  |  |  |  |  |  |  |  |
| --- | --- | --- | --- | --- | --- | --- | --- |
| Rank | Match Score | Redundant Motif | P-value | log P-value | % of Targets | % of Background | Motif file |
| 1 | 0.619 | A T G C C G A T A G T C G C T A T A C G G T C A T A C G T G A C | 1e-35 | -82.418587 | 21.84% | 19.57% | motif file (matrix) |
| 2 | 0.609 | A T G C G A T C A G T C G C T A A T C G T A G C T A C G T A G C A C G T A C T G | 1e-19 | -45.945667 | 22.97% | 21.26% | motif file (matrix) |
| 3 | 0.660 | A G T C A T G C A G T C C T G A A C T G A G T C A G T C A T G C | 1e-14 | -33.447274 | 4.90% | 4.19% | motif file (matrix) |
